# Transgenic strategies to confer resistance against viruses in rice plants

**DOI:** 10.3389/fmicb.2013.00409

**Published:** 2014-01-13

**Authors:** Takahide Sasaya, Eiko Nakazono-Nagaoka, Hiroaki Saika, Hideyuki Aoki, Akihiro Hiraguri, Osamu Netsu, Tamaki Uehara-Ichiki, Masatoshi Onuki, Seichi Toki, Koji Saito, Osamu Yatou

**Affiliations:** ^1^NARO Kyushu-Okinawa Agricultural Research CenterKoshi, Kumamoto, Japan; ^2^National Institute of Fruit Tree ScienceTsukuba, Ibaraki, Japan; ^3^National Institute of Agrobiological SciencesTsukuba, Ibaraki, Japan; ^4^Hokuriku Research Center, NARO Agricultural Research CenterJoetsu, Niigata, Japan; ^5^Graduate School of Agricultural and Life Sciences, The University of Tokyo BunkyoTokyo, Japan

**Keywords:** *Reoviridae*, *Tenuivirus*, RNA interference, transgenic rice, forage rice cultivar

## Abstract

Rice (*Oryza sativa* L.) is cultivated in more than 100 countries and supports nearly half of the world’s population. Developing efficient methods to control rice viruses is thus an urgent necessity because viruses cause serious losses in rice yield. Most rice viruses are transmitted by insect vectors, notably planthoppers and leafhoppers. Viruliferous insect vectors can disperse their viruses over relatively long distances, and eradication of the viruses is very difficult once they become widespread. Exploitation of natural genetic sources of resistance is one of the most effective approaches to protect crops from virus infection; however, only a few naturally occurring rice genes confer resistance against rice viruses. Many investigators are using genetic engineering of rice plants as a potential strategy to control viral diseases. Using viral genes to confer pathogen-derived resistance against crops is a well-established procedure, and the expression of various viral gene products has proved to be effective in preventing or reducing infection by various plant viruses since the 1990s. RNA interference (RNAi), also known as RNA silencing, is one of the most efficient methods to confer resistance against plant viruses on their respective crops. In this article, we review the recent progress, mainly conducted by our research group, in transgenic strategies to confer resistance against tenuiviruses and reoviruses in rice plants. Our findings also illustrate that not all RNAi constructs against viral RNAs are equally effective in preventing virus infection and that it is important to identify the viral “Achilles’ heel” gene to target for RNAi attack when engineering plants.

## INTRODUCTION

Rice (*Oryza sativa* L.), one of the most important grain crops, is grown worldwide, with more than 90% (650 million tons) in Asia, where it is consumed directly to supply 36% of the total calories consumed (Normile, 2008; Zeigler and Barclay, 2008). Of the 15 viruses damaging rice, 10 are a serious menace to rice production in Asia. In southern Vietnam during 2006–2007, more than 485,000 hectares of paddy fields were severely affected by infection with *Rice grassy stunt virus* (RGSV) or co-infection by RGSV and *Rice ragged stunt virus* (RRSV), resulting in the loss of 828,000 tons of rice valued at US$120 million and directly affecting millions of rice farmers (Cabauatan et al., 2009). The yield losses of rice caused by rice viruses are enormous. To ensure global food security for continuing population growth, controlling the various viruses that damage rice is vital.

The rice viruses encompass many types of viruses, e.g., double-stranded RNA (dsRNA) viruses such as *Rice dwarf virus* (RDV), *Rice black streaked dwarf virus* (RBSDV), and RRSV, negative-sense single-stranded RNA viruses, *Rice stripe virus* (RSV) and RGSV, a double-stranded DNA virus, *Rice tungro bacilliform virus*, and a positive-sense single-stranded RNA virus, *Rice tungro spherical virus*. Almost all these rice viruses are transmitted by leafhoppers and planthoppers, and some multiply in the insects and are transmitted transovarially, making their control more difficult (Hibino, 1996). These insect vectors are distributed widely in Asian countries and migrate long distances, even across the ocean (Kishimoto, 1971).

Recent advances in biotechnology should help solve these problems, and genetically engineering plants to have improved resistance against diseases and harmful insects is one of the most promising approaches. Based on the concept of pathogen-derived resistance, that the expression of various viral sequences is effective in preventing or reducing various plant virus infections, several strategies to confer resistance against viruses in plants have been developed over the last two decades (for reviews, see Sanford and Johnston, 1985; Baulcombe, 1996; Palukaitis and Zaitlin, 1997; Miller and Hemenway, 1998). Recently, viral RNA itself has been shown to be a potential trigger for resistance against viruses in transgenic plants, with the subsequent discovery of a novel, innate resistance in plants, known now as RNA interference (RNAi) or RNA silencing (for reviews, see Vazquez Rovere et al., 2002; Baulcombe, 2004, 2005; Voinnet, 2005; Ding and Voinnet, 2007).

RNA interference, an evolutionarily conserved process that is active in a wide variety of eukaryotic organisms, is a sequence-specific gene-silencing mechanism that is induced by dsRNA (for reviews, see Baulcombe, 2004, 2005; Voinnet, 2005). The dsRNA is diced into small interfering RNAs (siRNAs) of 21–24 nucleotides by an endonuclease called Dicer (Bernstein et al., 2001; Fusaro et al., 2006). These siRNAs are then incorporated into the RNA-induced silencing complex to guide degradation or translational repression in a sequence-specific manner. Via the expression of virus-specific dsRNA as hairpin structures, it is one of the relative easy and promising ways to render plants resistant against plant virus infection (Wang et al., 2000; Kalantidis et al., 2002; Bonfim et al., 2007).

Our successive attempts at conferring resistance against rice viruses have indicated that the RNAi constructs that target various viral genes are not equally effective in preventing virus infection; depending on the viral gene targeted, the levels of resistance have varied from complete resistance against a delay in symptom development or even an absence of resistance (Shimizu et al., 2009, 2011b). Thus, identifying the viral “Achilles’ heel” gene is important for choosing an appropriate target for the RNAi attack when engineering plants that are strongly resistant against virus infection. In this article, we review recent progress, mainly conducted by our research group, in transgenic strategies to confer resistance in rice plants against the rice viruses in the family *Reoviridae* and the genus *Tenuivirus* that cause serious problems for stable rice production in Asia. In addition, we discuss our recent most attempt to develop transgenic virus-resistant forage cultivars of rice that can be cultivated in fields to control virus diseases and reduce the population of viruliferous insect vectors.

## CONFERRING RESISTANCE AGAINST RICE-INFECTING REOVIRUSES

### RICE-INFECTING REOVIRUSES

Four dsRNA viruses, RDV, RBSDV, *Rice gall dwarf virus* (RGDV), and RRSV, occur in rice and threaten rice production in Asia. In addition, a new virus, Southern rice black-streaked dwarf virus (SRBSDV) that was first discovered in 2001 in Guangdong, China, has rapidly spread throughout central China and Vietnam, and has recently been found in northern China and Japan (Hoang et al., 2011; Wang et al., 2012).

On the basis of virion properties, insect vector specificity, viral genome organization, and viral sequence information, these five rice-infecting reoviruses are classified into three genera, *Oryzavirus*, *Phytoreovirus,* and *Fijivirus* of the family *Reoviridae* (Attoui et al., 2011). RDV and RGDV, belonging to the genus *Phytoreovirus*, are transmitted in a persistent manner by leafhoppers (*Nephotettix cincticeps*, *N. nigropictus*, and *Recilia dorsalis*). RRSV, belonging to the genus *Oryzavirus*, is transmitted in a persistent manner by the brown planthopper (*Nilaparvata lugens*). RBSDV and SRBSDV, belonging to the genus *Fijivirus*, are mainly transmitted by the small brown planthopper (*Laodelphax striatellus*) and the white-backed planthopper (*Sogatella furcimera* Horváth), respectively. All five of these reoviruses are propagative in their vectors, and RDV and RGDV can be transmitted from female adults to their progeny via eggs. RRSV occurs mainly in the tropical regions of Asia, such as Indonesia, Malaysia, the Philippines, Vietnam, and Thailand, where it causes serious problems for rice production. The other four viruses occur mainly in the subtropical regions of Asia, such as China, Japan, Korea, and Nepal (Hibino, 1996). Outbreaks of RBSDV have created serious problems not only for rice production but sometimes also for maize production in China and Japan (Ishii and Yoshimura, 1973; Li et al., 1999).

### VIRAL GENOME ORGANIZATION

**FIGURE 1:**
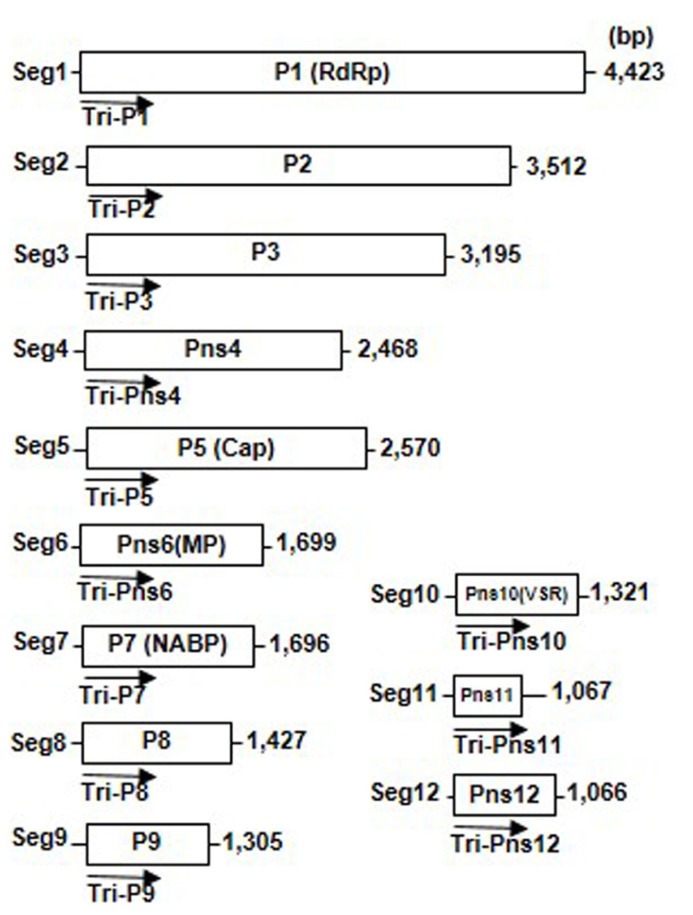
**Genome structure of *Rice dwarf virus* (RDV).** Map of segments 1 through 12 of the RDV genome. Lines represent the viral genomic double-stranded RNA segments; boxes denote genes encoded by RDV genes. Arrows indicate 500-bp RNA interference (RNAi) trigger sequences that were amplified from the 5′-proximal regions of each RDV gene and cloned into the RNAi trigger plasmids used to transform the plants. RdRp, RNA-dependent RNA polymerase; Cap, capping enzyme; MP, movement protein; VSR, silencing suppressor.

The genomes of the rice-infecting reoviruses consist of 10–12 segments of linear dsRNA and encode 10–12 proteins (**Figure 1**). The viral core particle appears to be formed by viral genomic dsRNA segments and at least four proteins (major core capsid, RNA-dependent RNA polymerase, capping enzyme, and nucleic acid binding protein). The core particles are surrounded by one or two outer capsid proteins and form a double-shelled, icosahedral particle approximately 60–80 nm in diameter. In addition to these structural proteins, the viral genomes encode two to five non-structural proteins, which form cytoplasmic inclusion bodies, known as viroplasms (Isogai et al., 1998; Omura and Yan, 1999; Attoui et al., 2011).

The reoviruses form distinctive structures in the cytoplasm of infected host cells at early stages of viral infection. Depending on the genus of the family *Reoviridae*, the cytoplasmic structures are called different names: viroplasms, viral factories, and viral inclusion bodies. However, these viral structures are thought to have essentially similar functions. Since the cytoplasmic structures are formed at early stages of viral infection in the host cells and contain many viral proteins, viral genomic segments, and virus particles, these cytoplasmic structures are considered to be the sites of viral RNA replication and packaging into progeny particles. Thus, the viroplasm-associated proteins may also play important roles in viral infection and proliferation at an early stage of viral replication (McNulty et al., 1976; Sharpe et al., 1982; Eaton et al., 1987; Touris-Otero et al., 2004). In the case of the rice-infecting reoviruses, proteins Pns6, Pns11, and Pns12 encoded by RDV segments 6, 11, and 12, Pns9 encoded by RGDV segment 9, NS10 by RRSV segment 10, and P9-1s by RBSDV and SRBSDV segment 9, have been confirmed as component proteins of the viroplasm (Isogai et al., 1998; Wei et al., 2006; Akita et al., 2011; Mao et al., 2013).

Regarding the adaptation of plant reoviruses to plants as hosts, two striking differences can be discerned between the animal-infecting reoviruses and their plant-infecting counterparts. The first is the ability of a plant reovirus to modify the plasmodesmata, the cytoplasmic channel through the plant cell walls, to facilitate systemic spread of infectious viral entities throughout the plant host. Most plant viruses have one or more genes for viral movement proteins that facilitate transport of the virus genome from an initially infected cell into neighboring cells through the plasmodesmata (for reviews, see Scholthof, 2005; Lucas, 2006; Taliansky et al., 2008). The viral movement proteins tend to be localized predominantly near or in the plasmodesmata. In addition, the viral movement protein of a certain virus can often complement the cell-to-cell movement of other distantly related or even unrelated viruses, even though viral movement proteins are highly variable in their amino acid sequences (Morozov and Solovyev, 2003; Xiong et al., 2008; Hiraguri et al., 2011, 2013). In the case of the rice-infecting reoviruses, Pns6 encoded by RDV segment 6 and P6 encoded by RRSV segment 6 were confirmed as a viral movement protein in experiments that localized the viral-encoded protein to the plasmodesmata and showed that the protein can restore cell-to-cell movement of a movement-defective virus (Li et al., 2004; Wu et al., 2010).

The second difference is the capacity of the plant reovirus to counteract RNAi, the innate antiviral defense system of plants and insects. RNAi involves a sequence-specific degradation that is induced by dsRNA molecules and can target transgenes as well as homologous endogenous genes. Because all RNA viruses replicate through the formation of dsRNA intermediates, these intermediates are potential targets for RNAi. To counteract the RNAi mechanism of their host, plant viruses have developed ways to evade or neutralize this response. The most common way is to encode the so-called RNA silencing suppressor proteins (VSR). A simple and elegant assay for RNAi suppressor activity that involves a combination of green fluorescent protein (GFP)-silenced reporter plants and *Agrobacterium*-based transient transformation has been developed (Voinnet et al., 2000; Bucher et al., 2003; Baulcombe, 2004).

A large number of RNA silencing suppressor proteins have been identified from numerous plant viruses (Pruss et al., 1997; Brigneti et al., 1998; Voinnet et al., 2000; Dunoyer et al., 2002; Pfeffer et al., 2002; Bucher et al., 2003; Zhang et al., 2005). In the case of rice-infecting reoviruses, Pns10 encoded by RDV segment 10 and Pns11 encoded by RGDV segment 11, have been shown to have RNA silencing suppressor activity (Cao et al., 2005; Zhang et al., 2005; Liu et al., 2008).

### RESISTANCE AGAINST RICE DWARF VIRUS IN TRANSGENIC RICE

RNA interference has been an important tool to render plants resistant against virus infections. During our work to develop resistance against RDV in rice plants by introducing dsRNAs of a 500-bp fragment in the 5′-proximal regions of each viral genes as the viral target genes (**Figure 1**), the degrees of resistance that were conferred to rice differed (Shimizu et al., 2009; Sasaya et al., 2013). The degrees of resistance against RDV infection differed, depending on the viral target genes, from almost immune (i.e., no symptom development or virus amplification), moderate resistance, to no resistance (**Table 1**).

**Table 1:** Degree of resistance against *Rice dwarf virus* (RDV) infection in transgenic rice plants induced by different RNAi-targets of RDV genes^a^.

Target gene for	Location/putative function^b^	GenBank accession	Resistance^c^
P1	Core particle/RNA polymerase	D90198	Strong
P2	Outer particle/vector transmission	AB263418	Absent
P3	Core particle/major core capsid	X54620	Moderate
Pns4	Cytoplasmic fibril/intracellular movement	X54622	Strong
P5	Core particle/capping enzyme	D90033	Absent
Pns6	Viroplasm/movement protein	M91653	Immune
P7	Core particle/nucleic acid binding	D10218	Absent
P8	Outer particle/major outer capsid	D10219	Immune
P9	Outer particles/unknown	D10220	Absent
Pns10	Tubule structure/silencing suppressor	D10221	Absent
Pns11	Viroplasm/unknown	D10249	Strong
Pns12	Viroplasm/unknown	D90200	Immune

a To evaluate any resistance to RDV infection, more than 30 rice plants from three independent lines of transgenic plants were exposed to approximately 10 viruliferous RDV-carrying viruliferous leaf hopper per plant for 1 day.

b Suzuki etal. (1990a,b, 1991, 1992a,b), Suzuki (1993), Uyeda etal. (1994), Zhong etal. (2003), Li etal. (2004), Cao etal. (2005).

c Immune, no symptoms developed, and no virus was detected by ELISA in inoculated rice plants through harvest; Strong, weak symptoms developed but were delayed for 2–4 weeks, but growth was almost the same as for mock-inoculated rice plants; Moderate, typical symptoms developed but were delayed 2–4 weeks, and growth was slightly stunted after RDV infection; Absent, typical symptoms developed, as severe as those of RDV-infected non-transgenic rice plants.

**FIGURE 2:**
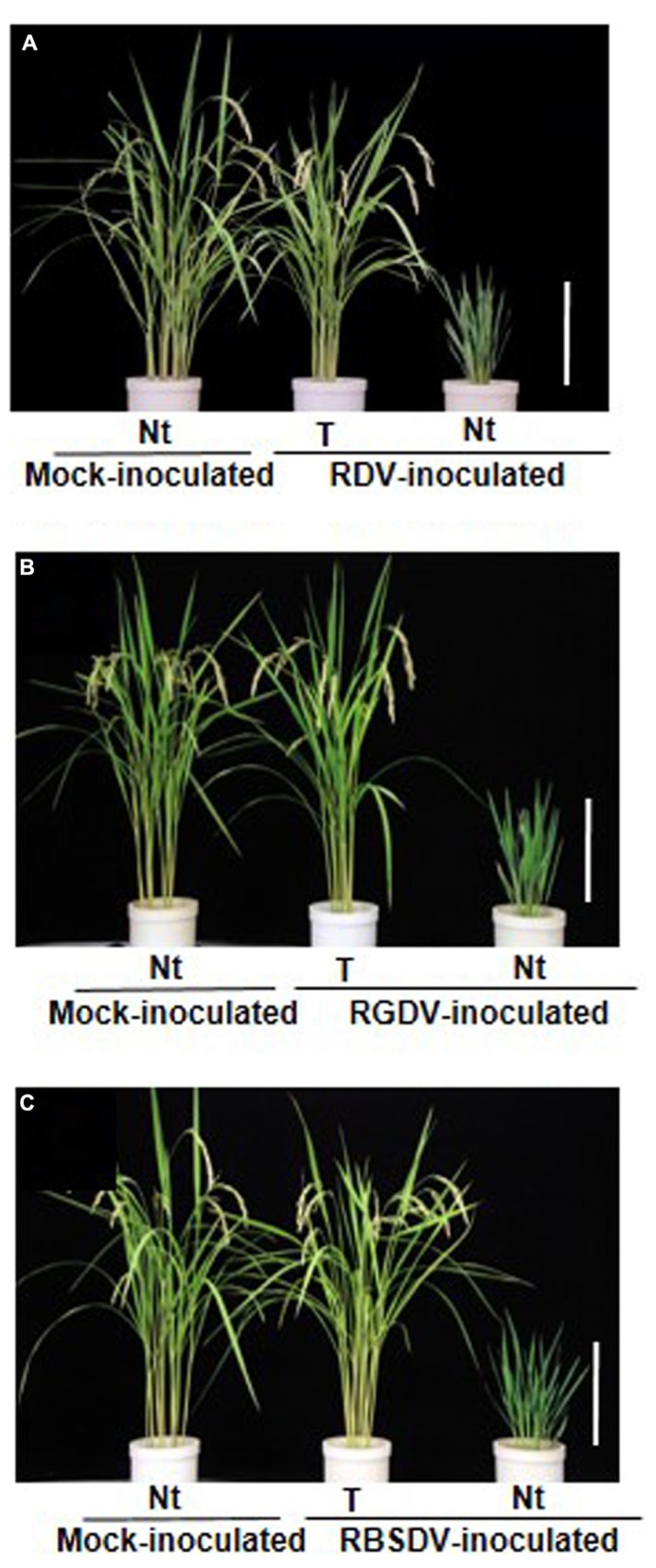
**Transgenic rice plants enhanced resistance against three rice-infecting reoviruses. (A)** Phenotype of transgenic rice plants (cv. Nipponbare) that harbor the RNAi trigger sequence targeting the *Rice dwarf virus* (RDV) gene for Pns12 (from Shimizu et al., 2009). **(B)** Phenotype of transgenic rice plants that harbor the RNAi trigger sequence targeting the *Rice gall dwarf virus* (RGDV) gene for Pns9 (from Shimizu et al., 2012). **(C)** Phenotype of transgenic rice plants that harbor the RNAi trigger sequence targeting the *Rice black streaked dwarf virus* (RBSDV) gene for P9-1 (from Shimizu et al., 2011a). Ten-day-old transgenic rice seedlings were exposed to approximately 10–15 viruliferous vector insects per plant for 1 day and evaluate plant response to virus infection at 4 months after virus inoculation. Rice plants in pots from left to right: mock-inoculated non-transgenic rice plants (Nt) exposed to virus-free insect vectors, showing normal growth; virus-inoculated transgenic rice plants (T), showing healthy growth and fertility after inoculation; virus-inoculated non-transgenic rice plant (Nt), showing typical symptoms caused by RDV, RGDV or RBSDV infection. Bar, 30 cm.

The transgenic rice plants with the introduced RNAi construct targeting the RDV gene for Pns 6 (viroplasm associated protein and movement protein), P8 (major outer capsid), and Pns12 (viroplasm associated protein) were almost immune to RDV infection. There were no apparent differences in morphology or growth, based on plant height, number of tillers, and rice grain yields between inoculated and mock-inoculated rice plants (**Figure 2A**). The transgenic plants did not contain detectable amounts of the virus through harvest as determined by ELISA. In contrast, transgenic plants with an introduced construct for P2 (outer capsid), P5 (capping enzyme), P7 (nucleic acid binding protein), P9 (outer capsid), or Pns10 (silencing suppressor) did not develop any resistance against RDV; symptoms developed at the same rate and severity as those in the susceptible non-transgenic control plants.

By analyzing the effects of potential target sequences in each of the coding genes in the RDV genome, we found transgenic plants that harbored the RNAi constructs targeting the genes for Pns6, P8, or Pns12 were completely resistant against the RDV infection, suggesting that these proteins are key components at the early stages of viral proliferation. Reiterating the functions of these proteins, P8 is a major outer capsid, and Pns6 and Pns12 are component proteins of the viroplasm, and Pns6 also functions as a viral movement protein. Thus, when the plants inhibited the expression of the viral major outer capsid, viroplasm-associated protein and viral movement protein via RNAi, they developed the strongest resistance against RDV infection.

By contrast, transgenic plants that harbored the RNAi construct specific for the genes for P2 and Pns10 were susceptible to RDV. Considering that when RDV is maintained in rice plants for a long period, non-sense mutations gradually accumulate in RDV segments 2 and 10, resulting in a decrease in the expression of these proteins and complete loss of insect-transmissibility, these proteins might not be essential for viral proliferation in rice plants but instead function in the insect vectors (Pu et al., 2011). Thus, transgenic plants with the introduced RNAi constructs targeting the RDV genes for P2 and Pns10 seem not to have induced resistance against RDV infection.

At any rate, not all RNAi constructs against RDV genes are equally effective in preventing viral infection. The genes for the major outer capsid, viroplasm associated proteins, and viral movement protein can be considered the “Achilles’ heel” of the rice-infecting reoviruses and be targeted for RNAi attack for engineering resistance in plants.

### CONFERRING STRONG RESISTANCE AGAINST OTHER REOVIRUSES

The results discussed in the previous section brought the idea that the transgenic rice plants inhibited the expression of other reoviral gene for the viroplasm associated protein showed complete resistance against their other reoviruses. Because RGDV Pns9 and RBSDV P9-1 are the functional orthologs of the viroplasm associated protein of RDV, the genes for Pns9 and P9-1 might be thought the appropriate targets for suppressing the proliferation of RGDV and RBSDV, respectively, in infected rice plants (Isogai et al., 1998; Akita et al., 2011). To make clear that these target genes induce complete resistance against these rice-infecting reoviruses by RNAi, the rice plants (cv Nipponbare), which have been introduced the RNAi trigger plasmids transcribed into dsRNAs of a 500-bp fragment in the 5′-proximal regions of the genes for Pns9 and P9-1 were evaluated any subsequent resistance against RGDV or RBSDV infections, respectively (Shimizu et al., 2011a, 2012).

All transgenic plants with the introduced RNAi trigger construct of the RGDV gene for Pns9 were asymptomatic and continued to be symptom-free until harvest (ca. 4 months), whereas growth of the RGDV-infected non-transgenic rice plants was severely stunted and small galls had developed along the leaf veins on the abaxial surface of leaves and on the outer surface of sheaths by 4 weeks post-inoculation. The transgenic rice plants that remained asymptomatic after challenge with RGDV, were almost immune to RGDV infection because the transgenic plants did not contain detectable amounts of the virus, as determined by ELISA. In addition, no apparent differences in morphology or growth, based on plant height, number of tillers, and rice grain yields were observed between transgenic and mock-inoculated non-transgenic rice plants (**Figure 2B**).

Similarly, the transgenic rice plants that harbored the RNAi trigger plasmid that inhibits the expression of the RBSDV gene for P9-1 were completely resistant against RBSDV infection and remained asymptomatic with no virus detectable by ELISA after challenge with RBSDV. In contrast, the RBSDV-infected non-transgenic rice plants were severely stunted with darkened leaves, twisted leaf tips, split leaf margins, and waxy white-to-black galls along the veins on the adaxial surface of leaf blades and the adaxial surface of sheaths by 4 weeks post-inoculation (**Figure 2C**).

These findings further confirmed evidence that the genes for the viroplasm associated proteins are the viral “Achilles’ heel” for RNAi attack and that these genes can be targeted to confer strong resistance against plant-infecting reoviruses in transgenic rice plants. Furthermore, our strategy to interfere with the expression of viroplasm-associated proteins can induce strong resistance and should be effective for controlling other rice-infecting reoviruses of important crop plants such as RRSV and SRBSDV. The RRSV gene for NS10 and the SRBSDV gene for P9-1 are promising genes to target.

## CONFERRING RESISTANCE AGAINST RICE-INFECTING TENUIVIRUSES

### RICE-INFECTING TENUIVIRUSES

Three tenuiviruses, RGSV, RSV, and *Rice hoja blanca virus* (RHBV) are known to cause severe damage to rice. RSV is transmitted in a persistent manner by the small brown planthopper and induces significant economic losses in temperate regions of East Asia, especially in China, Japan, and Korea. RGSV, transmitted to rice plants in a persistent manner by the brown planthopper, has become a serious problem for rice production in South, Southeast, and East Asian countries (Hibino, 1996). RHBV is propagatively transmitted by the planthopper *Tagosodes orizicolus* and occurs in Central and South America, the Caribbean, and the southern United States (Falk and Tsai, 1998). RSV and RHBV are transmitted transovarially to progeny at high rates, but RGSV is not (Hibino, 1996; Falk and Tsai, 1998).

### VIRAL GENOME ORGANIZATION

**FIGURE 3:**
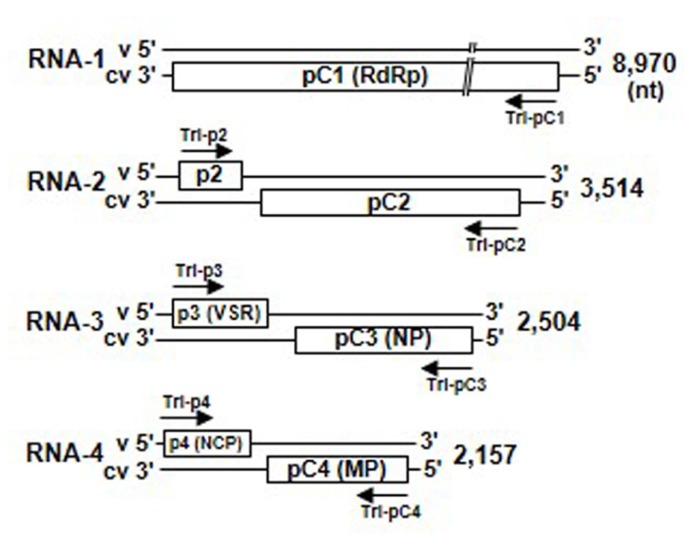
**Genome structure of *Rice stripe virus* (RSV).** Map of RNAs 1 through 4 of the RSV genome. Upper and lower lines represent the virion-sense RNA segments and virion complementary-sense RNA segments, respectively, and boxes denote genes encoded by RSV genes. Arrows indicate 500-bp RNA interference (RNAi) trigger sequences that were amplified from the 5′-proximal regions of each RSV gene and cloned into the RNAi trigger plasmid for plant transformation. MP, movement protein; NCP, major non-capsid protein; NP, nucleocapsid protein; RdRp, RNA-dependent RNA polymerase; VSR, silencing suppressor.

Virus particles of the tenuiviruses are thin filaments 3–10 nm in diameter and may be composed of a single nucleocapsid protein and four to six single-stranded RNA segments with positive (virion-sense) and negative (virion complementary-sense) polarities (**Figure 3**; Shirako et al., 2011). The genomes of RSV and RHBV consist of four ssRNA segments, designated RNAs 1–4 in order of decreasing molecular mass, and have seven genes. The first RNA segment has negative polarity, and the other three are ambisense. The RGSV genome consists of six ssRNA segments, all of which are ambisense, and includes 12 genes. The viral mRNAs are transcribed from each viral gene by the cap-snatching mechanism (Ramirez et al., 1995; Shimizu et al., 1996).

The unusual genome organization and replication strategy of tenuiviruses has so far prevented the development of an infectious clone system for the virus. The lack of a reverse genetics system for any viruses in this genus has been an obstacle for functional studies of virus-encoded proteins using standard mutagenesis. The functions of several virus-encoded proteins have, however, been predicted (**Table 2**). The pC1 encoded in the first RNA segment of the *Tenuivirus* genome contains the major RNA-dependent RNA polymerase modules and RNA-dependent RNA polymerase activity (Toriyama, 1986; Toriyama et al., 1994, 1998; Nguyen et al., 1997). The pC2 is encoded in the virion-complementary sense of the second RNA segment, has stretches of weak but significant amino acid similarity with amino acids of the glycoproteins of phleboviruses and may be evolutionarily linked with the membrane-associated glycoprotein (Takahashi et al., 1993). The p3 protein encoded in the virion-sense of RNA-4 of RSV and RHBV functions as the viral silencing suppressor (Bucher et al., 2003; Hemmes et al., 2007; Schnettler et al., 2008; Xiong et al., 2009). The pC3 protein encoded in the virion-complementary sense of RNA-3 of RSV and RHBV and pC5 encoded in the virion-complementary sense of RGSV RNA-5 are nucleocapsid proteins and major components of the thin filamentous particles (Zhu et al., 1991; de Miranda et al., 1994; Toriyama et al., 1997). The p4 protein encoded in the virion-sense strand of RNA-4 of RSV and RHBV and p6 encoded by the virion-sense of the RGSV RNA-6 accumulate in large amounts and form crystalline inclusion bodies in virus-infected rice plants, but their functions are unknown (Koganezawa, 1977; Falk et al., 1987; Hayano et al., 1990; Miranda and Koganezawa, 1995). The pC4 protein encoded in the virion-complementary sense of RNA-4 of RSV and pC6 encoded in the virion-complementary sense of the RNA-6 of RGSV are viral movement proteins; their proteins accumulate close to the cell walls of infected host cells and facilitate intercellular transport of movement-defective viruses (Xiong et al., 2008; Hiraguri et al., 2011).

**Table 2:** Degree of resistance against *Rice stripe virus* (RSV) infection in transgenic rice plants induced by different RNAi-targets of RSV genes^a^.

Target gene for	Location/putative function^b^	GenBank accession	Resistance^c^
pC1	RNA polymerase	D31879	Strong
p2	Unknown	D13176	Moderate
pC2	Glycoprotein-like	D13176	Absent
p3	Silencing suppressor	X53563	Moderate
pC3	Nucleocapsid protein	X53563	Immune
p4	Crystalline inclusion	D10979	Absent
pC4	Movement protein	D10979	Immune

a To evaluate any resistance to RSV infection, more than 30 rice plants from three independent lines of transgenic plants were exposed to approximately 15 viruliferous RSV-carrying viruliferous small brown hopper per plant for 1 day.

b Kakutani etal. (1990), Zhu etal. (1991), Takahashi etal. (1993), Toriyama etal. (1994), Xiong etal. (2008, 2009).

c Immune, no symptoms developed, and no virus was detected by ELISA in inoculated rice plants through harvest; Strong, weak symptoms developed but were delayed for 2–4 weeks, but growth was almost the same as for mock-inoculated rice plants; Moderate, typical symptoms developed but were delayed 2–4 weeks, and growth was slightly stunted after RSV infection; Absent, typical symptoms developed, as severe as those of RSV-infected non-transgenic rice plants.

### RESISTANCE AGAINST RICE STRIPE VIRUS IN TRANSGENIC RICE

To identify the most appropriate target genes for induction of strong resistance against tenuiviruses by RNAi, we have generated transgenic rice plants that inhibited the expression of one of the various coding genes in the RSV genome. Seven RNAi trigger plasmids that would be transcribed into dsRNAs of the 5′-proximal regions of each gene in the RSV genome were constructed and introduced into the rice plants (cv. Nipponbare; **Figure 3**) for evaluating resistance against RSV infection (Shimizu et al., 2011b).

**FIGURE 4:**
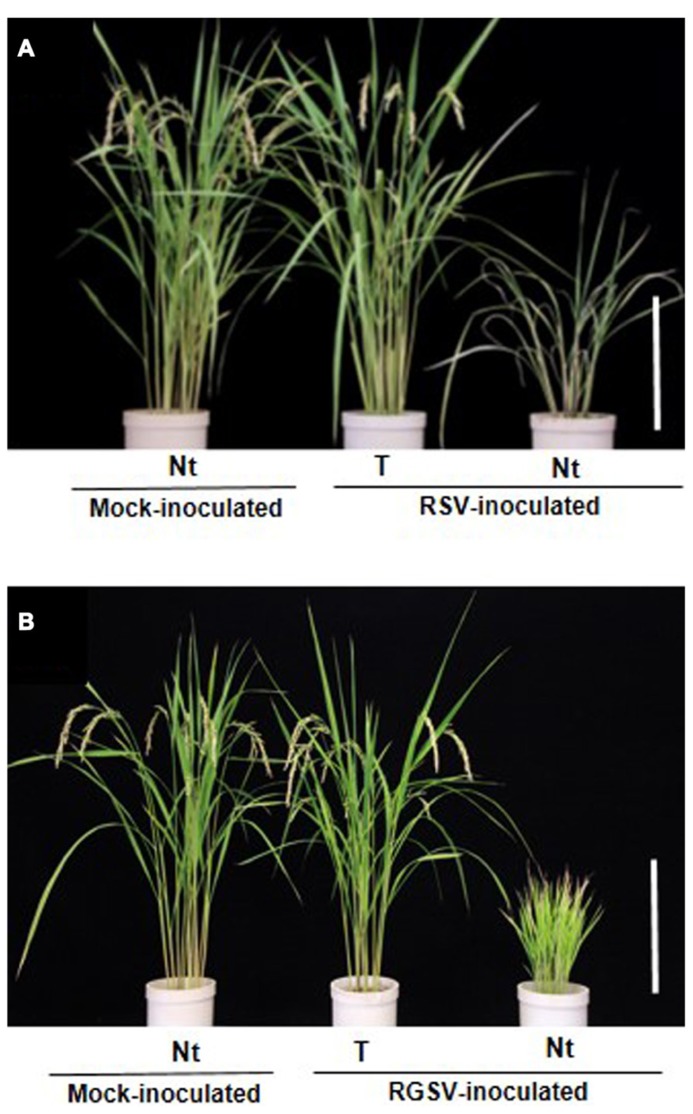
**Transgenic rice plants with enhanced resistance against two tenuiviruses. (A)** Phenotype of transgenic rice plants (cv. Nipponbare) that harbored the RNAi trigger sequence targeting the *Rice stripe virus* (RSV) gene for pC3 (from Shimizu et al., 2011b). **(B)** Phenotype of transgenic rice plants that harbored the RNAi trigger sequence targeting the *Rice grassy stunt virus* (RGSV) gene for pC5 at 4 months after RGSV inoculation (from Shimizu et al., 2013). Ten-day-old transgenic rice seedlings were exposed to approximately 10–15 viruliferous vector insects per plant for 1 day and evaluate plant response to virus infection. Rice plants in pots from left to right: mock-inoculated non-transgenic rice plants (Nt) exposed to virus-free insect vectors, showing normal growth; virus-inoculated transgenic rice plants (T), showing healthy growth and fertility after inoculation; virus-inoculated non-transgenic rice plant (Nt), showing typical symptoms caused by RSV or RGSV infection. Bar, 30 cm.

The transgenic rice plants with the introduced RNAi trigger construct of the various individual RSV genes exhibited varying degrees of resistance against RSV infection (**Table 2**). The transgenic plants with the RNAi trigger plasmids of RSV genes for pC3 (nucleocapsid protein) and pC4 (movement protein) were conferred near immunity to RSV infection because the virus was not amplified in the transgenic rice plants through harvest (ca. 4 months). Furthermore, morphology and growth, based on plant height, number of tillers, and rice grain yields apparently did not differ between the transgenic and mock-inoculated non-transgenic rice plants (**Figure 4A**). In contrast, the transgenic plants with the introduced pC2 or p4 did not have any resistance against RSV infection; symptoms developed at the same rate and severity as in the susceptible non-transgenic control plants. Transgenic rice plants with the p2 and p3 constructs exhibited moderate resistance against RSV infection; typical symptoms were induced but their appearance was delayed for 2–4 weeks, and plant growth was moderately stunted by RSV infection.

### CONFERRING STRONG RESISTANCE AGAINST RICE GRASSY STUNT VIRUS

Because the genes for the nucleocapsid protein and movement protein were appropriate targets for RNAi to confer complete resistance against RSV infection, transgenic rice plants that expressed dsRNAs of the RGSV genes for pC5 and pC6, the functional orthologs of pC3 and pC4 of RSV, respectively (Hiraguri et al., 2011) were generated with the expectation that they would be appropriate targets for completely suppressing the proliferation of RGSV in infected rice plants (Shimizu et al., 2013).

All the transgenic plants with the introduced RNAi trigger constructs for pC5 and pC6 were asymptomatic at 4 weeks post-inoculation, in contrast to the typical severe stunting of plant growth with profuse tillering of all infected non-transgenic plants, and they continued to be symptom-free and with no detectable amounts of the virus until harvest (ca. 4 months). In addition, no apparent differences in morphology or growth, based on plant height, number of tillers, and rice grain yields were observed between transgenic and mock-inoculated non-transgenic rice plants (**Figure 4B**). These results indicated that the RGSV genes for the nucleocapsid protein and movement protein are the viral “Achilles’ heel” to target for RNAi attack and provided further evidence that targeting these genes are effective in conferring strong resistance against tenuiviruses in transgenic rice plants. Our strategy for interfering with the expression of the nucleocapsid protein and movement protein should also be effective for controlling other tenuiviruses. For example, the genes for pC3 and pC4 in RHBV, which causes economically important rice crop plants in Central and South America, are promising candidates to confer strong resistance against RHBV infection in rice plants.

## VIRUS-RESISTANT TRANSGENIC FORAGE RICE CULTIVAR: PRACTICAL APPLICATION

From the 1960s on, the production of livestock feed in Japan has decreased dramatically to only 26% of the total consumed by 2012, critically impairing the ability to maintain stable production of livestock (Annual Governmental Report about Agricultural Trends 2012, available at ). To increase Japan’s self-sufficiency for livestock feed, the production of forage rice cultivars is strongly promoted by the Japanese government, and significant progress in research on forage rice cultivars has resulted in several new rice cultivars that produce large gross weights.

As global temperatures have increased, viruliferous insects that can transmit rice viruses are also on the increase (Shiba, 2012). Damage caused by rice viruses and their insect vectors has become a serious problem for both edible and forage rice cultivars in Japan. The risk of outbreaks of rice viruses, especially RSV and RDV, which have caused serious yield loss in rice production before the 1960s (Toriyama, 2010), is increasing. The use of insecticides to control the vector insects is one of the most effective methods of protecting rice plants from virus infection. However, the high cost of insecticide application is a major burden to rice growers, and cost-prohibitive for forage rice growers, who are forced to reduce the cost of rice production as much as possible. Genetic resistance against rice viruses or their insect vectors is also one of the most effective methods of protecting rice plants from virus infection. Although several rice genes confer resistance against RSV infection, the resistance is only partial (Noda et al., 1991). Furthermore, there are no reports, to the best of our knowledge, of naturally occurring genes that confer resistance against RDV infection.

**FIGURE 5:**
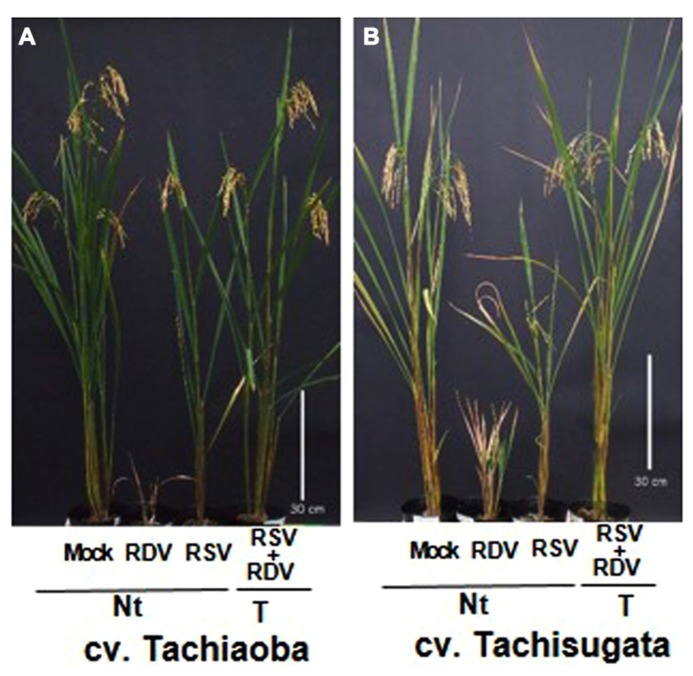
**Transgenic forage rice cultivars inoculated with *Rice stripe virus* (RSV) and *Rice dwarf virus* (RDV) (from Sasaya et al., 2013)**. Phenotypes of transgenic forage rice cultivars Tachiaoba **(A)** and Tachisugata **(B)** that harbored the RNAi trigger sequence targeting the RSV gene for pC3 and the RDV gene for Pns12 at 5 months after RDV and RSV inoculation. Ten-day-old transgenic forage rice seedlings were exposed to approximately 15 RSV-carrying viruliferous small brown hoppers and 10 RDV-carrying leaf hoppers per plant for each 1 day, and evaluate plant response to infection with RSV and RDV at 4 months after virus inoculation. The forage rice cultivars in pots from left to right are: mock-inoculated non-transgenic forage rice cultivars (Nt) exposed to virus-free insect vectors, showing normal growth; RDV-inoculated non-transgenic forage rice cultivars (Nt), showing typical symptoms caused by RDV infection; RSV-inoculated non-transgenic forage rice cultivars (Nt), showing typical symptoms caused by RSV infection; RDV+RSV-inoculated transgenic forage rice cultivars, showing healthy growth and fertility. Bar, 30 cm.

For the reasons that we have discussed, the use of RNAi to genetically engineer plants with improved resistance against these viruses has been considered one of the most promising approaches to solve these virus problems. To develop transgenic forage cultivars with strong resistance against RSV and RDV, we generated an RNAi trigger plasmid by introducing a fused chimeric gene composed of 500-bp fragments from the 5′-proximal region for the gene for RSV pC3 and for RDV Pns12. The plasmid was then introduced into two popular forage rice cultivars, Tachisugata and Tachiaoba, and the T1 generation plants were used to evaluate plant response to infection with RSV and RDV (**Figure 5**; Sasaya et al., 2013).

All the transgenic forage cultivar plants with the introduced RNAi trigger construct for RSV pC3 and the RDV Pns12 continued to be symptom-free and did not contain detectable amounts of these viruses through harvest (ca. 5 months), whereas the RSV- and the RDV-infected non-transgenic forage cultivar plants developed typical severe symptoms by 4 weeks post-inoculation. These transgenic forage cultivar plants were almost immune to RSV and RDV infections. In addition, no apparent differences in morphology or growth, based on plant height, number of tillers, and grain yield, were observed between transgenic and mock-inoculated non-transgenic forage cultivar plants (**Figure 5**). These results indicated that targeting the genes for the nucleocapsid protein and the viroplasm associated protein are effective in conferring strong resistance against the plant-infecting tenuivirus and reovirus, respectively, not only in the edible rice cultivar Nipponbare but also in the forage rice cultivars Tachisugata and Tachiaoba. Furthermore, simultaneously using the fused chimeric gene construct for the nucleocapsid protein and the viroplasm associated protein is effective in conferring strong resistance against both the viruses.

## CONCLUSION

Rice viruses cause significant economic losses in rice production in South, Southeast, and East Asian countries. The use of insecticides against insect vectors is one of the most effective approaches to prevent damage and yield loss from rice viruses, but the high cost is a major burden on rice growers, and the continuous usage of certain insecticides is likely to result in the insect vectors developing resistance against the insecticide (Brogdon and McAllister, 1998; Wang et al., 2007). Exploitation of genetic resistance against the rice viruses is another common approach to control rice plants from viral infection. However, a few rice cultivars/lines that show resistance against the viruses have been described (Noda et al., 1991; Azzam and Chancellor, 2002; Zhang et al., 2011).

The use of RNAi is an effective, more promising method to confer strong resistance against rice viruses. When developing transgenic rice plants with strong resistance against rice viruses, it is important to target viral genes that play important roles in viral infection and proliferation at an early stage of viral replication. Furthermore, the spectrum of RNA-mediated virus resistance is generally restricted to viral strains with greater than ≈90% sequence identities with the introduced transgenes (Jones et al., 1998; Bau et al., 2003; Bucher et al., 2006; Hassani-Mehraban et al., 2009). When we compared the nucleotide sequences of the gene for the nucleocapsid protein between our strain and all the strains of RSV that are available in DDBJ/EMBL/GeneBank, the 500 bp of the RNAi trigger sequence used for cloning and transformation in our transgenic experiment, share 95.0–99.2% nucleotide sequence identities with those of other RSV strains. Thus, our transgenic plants seem to possess a potentially durable, broad-spectrum resistance against heterologous strains of RSV originating from different regions.

These transgenic techniques to confer strong resistance against virus infection are a promising approach to control viral diseases and should contribute greatly to ensuring a stable food supply in Asian countries burdened with viral disease. It is important to advance research on the development of virus-resistant transgenic rice plants, although there are many obstacles that must be overcome to actually cultivate such virus-resistant transgenic rice plants in these areas. Other than transgenic papaya resistant against papaya ring spot virus (Bau et al., 2003; Mendoza et al., 2008), no transgenic crops intended for human consumption have been widely accepted, so they are not yet cultivated in the world. The primary reason is that consumers are afraid to eat transgenic foods. Developing a virus-resistant forage rice cultivar is, however, one way to gain acceptance of the use of transgenic crops because forage rice cultivars are not edible and transgenic forage crops such as BT-corn have generally been accepted for field cultivation. These transgenic forage cultivar plants should also play a role in decreasing the viruliferous insect populations, and hence, decreasing the incidence of viral diseases not only of forage rice cultivars but also of edible rice cultivars.

## Conflict of Interest Statement

The authors declare that the research was conducted in the absence of any commercial or financial relationships that could be construed as a potential conflict of interest.
